# Heat-inactivated *Lacticaseibacillus paracasei* N1115 alleviates the damage due to brain function caused by long-term antibiotic cocktail exposure in mice

**DOI:** 10.1186/s12868-022-00724-w

**Published:** 2022-06-26

**Authors:** Yujie Zhang, Huijing Liang, Yimie Wang, Ruyue Cheng, Fangfang Pu, Yang Yang, Jinxing Li, Simou Wu, Xi Shen, Fang He

**Affiliations:** 1 Department of Nutrition and Food Hygiene, West China School of Public Health and West China Fourth Hospital, Sichuan University, No. 16, 3rd section, South Renmin Road, Wuhou District, Chengdu, 610041 Sichuan People’s Republic of China; 2 Department of Clinical Nutrition, West China Hospital, Sichuan University, Chengdu, Sichuan People’s Republic of China

**Keywords:** Antibiotic, Cognitive function, Microbiota–gut–brain axis, Paraprobiotic

## Abstract

Critical development period of intestinal microbiota occurs concurrently with brain development, and their interaction is influenced by the microbiota–gut–brain axis. This study examined how antibiotics exposure affected gut microbiota and brain development and analyzed the possible benefits of heat-inactivated *Lacticaseibacillus paracasei* N1115 (N1115). Thirty neonatal male mice were randomly divided into three groups and treated with sterilized water (control), an antibiotic cocktail (Abx), or antibiotics plus heat-inactivated N1115 (Abx + N1115) for 84 days. We found that while the mRNA levels of GABA_Aα1_, GABA_b1_, and glucocorticoid receptor (GR) in the hippocampus and brain-derived neurotrophic factor (BDNF), GABA_Aα1_, GABA_b1_, and nerve growth factor (NGF) in the prefrontal cortex were higher, the mRNA levels of 5-HT_1A_ were lower in the Abx group. The Abx + N1115 group had lower mRNA levels of GABA_Aα1_, GABA_b1_, and GR in the hippocampus and BDNF, GABA_b1_, and NGF in the prefrontal cortex than the Abx group. The latency period was longer in the Morris water maze test while longer rest time was seen in tail suspension test in the Abx group than the control and Abx + N1115 groups. In the open field test, the moving time and distance of the Abx group were reduced. Further, the alpha-diversity indexes of the Abx and Abx + N1115 groups were significantly lower than the control. Further, long-term exposure to antibiotics disrupted the intestinal microbiota as evidenced by decreased *Bacteroides*, *Firmicutes*, and *Lactobacillus*, and increased *Proteobacteria* and *Citrobacter*. However, N1115 significantly decreased the abundance of *Citrobacter* when compared with those in the Abx group. These results indicate that antibiotics can substantially damage the intestinal microbiota and cognitive function, causing anxiety and depression, which can be alleviated by heat-inactivated N1115 via modulation of the microbiota–gut–brain axis.

## Introduction

The gut microbiota structure is dynamic and has inherent characteristics at different ages [1, 2]. The first 2 or 3 years of life is crucial for colonization of the gut microbiota, which is low in abundance and diversity during this period, and it is directly affected by many factors, such as the delivery and feeding modes, and use of antibiotics or probiotics. During childhood, the gut microbial diversity increases and becomes 40–60% similar to an adult [3], after which its declines [4, 5].

The brain function development is similar to that of gut microbiota as it rapidly develops from late pregnancy to 24 months postpartum, coinciding with gut microbiota colonization. During childhood, cognitive abilities begin to develop with learning abilities reaching its peak in adolescence. After adulthood, brain function reaches maturity with further learning. In the elderly, apoptosis of nerve cells and development of certain diseases with impaired brain function such as Alzheimer’s disease (AD) and Parkinson’s disease (PD) may occur [6, 7]. Hence, the critical periods for brain function development and gut microbiota overlap. Consequently, the gut microbiota structure in patients with brain function-related diseases differs from healthy people [8–10]. Regulating gut microbiota can provide symptomatic relief against these diseases [11].

Although how probiotics modulate the microbiota and their health benefits have been widely researched, their application is still challenging. Firstly, it is difficult to keep these probiotics during production, transportation, and storage. Studies have shown that certain probiotic strains found in yogurt lose their activity even when stored at a low temperatures [12]. Secondly, people with weakened immune function might face safety issues, for example, probiotic use of *Lactobacillus rhamnosus* GG is associated with bacteremia in children with short gut syndrome and adults with severe active ulcerative colitis [13–15]. Therefore, paraprobiotics, which are inactivated probiotics made of non-viable microbial cells (intact or broken) or crude cell extracts, have attracted attention as they are beneficial to the organism when administered in adequate amounts [16]. Several in vivo and in vitro studies have shown that paraprobiotics have immunomodulatory effects [17, 18]. Moreover, antibiotics do not interfere with their positive effects in the host as they are non-bacterial. *Lacticaseibacillus paracasei* N1115 (N1115) can prevent respiratory infections and alcoholic liver cirrhosis in elderly rodents [19–21]. Our previous studies showed that heat-inactivated N1115 might alleviate the short-term antibiotics-induced abnormal expression of brain-derived neurotrophic factor (BDNF), GABA and 5-HT receptors in the hippocampus of neonatal mice through gut-brain axis [22]. However, it is unclear whether heat-inactivated N1115 could also protect adult mice from long-term antibiotics-induced abnormalities in cognitive function and emotion. Therefore, we focused on whether paraprobiotics, like heat-inactivated N1115, can relieve brain dysfunction caused by antibiotics and its possible mechanism.

## Materials and methods

### Animals

Eighteen pregnant Kunming mice (from the Institute of Laboratory Animal Sciences of the Sichuan Academy of Medical Sciences and Sichuan Provincial People’s Hospital, Sichuan, China) were housed in a specific pathogen-free facility with free access to water and food in an environment with a12-h light/dark cycle, humidity of 55 ± 5%, and temperature of 22 ± 1 °C. The newborn pups were randomly divided into three groups (n = 6): a control group, an antibiotics cocktail (Abx) and an antibiotics + *L. paracasei* N1115 (Abx + N1115) groups [22].

#### Antibiotic and probiotic treatment

**Fig. 1:**
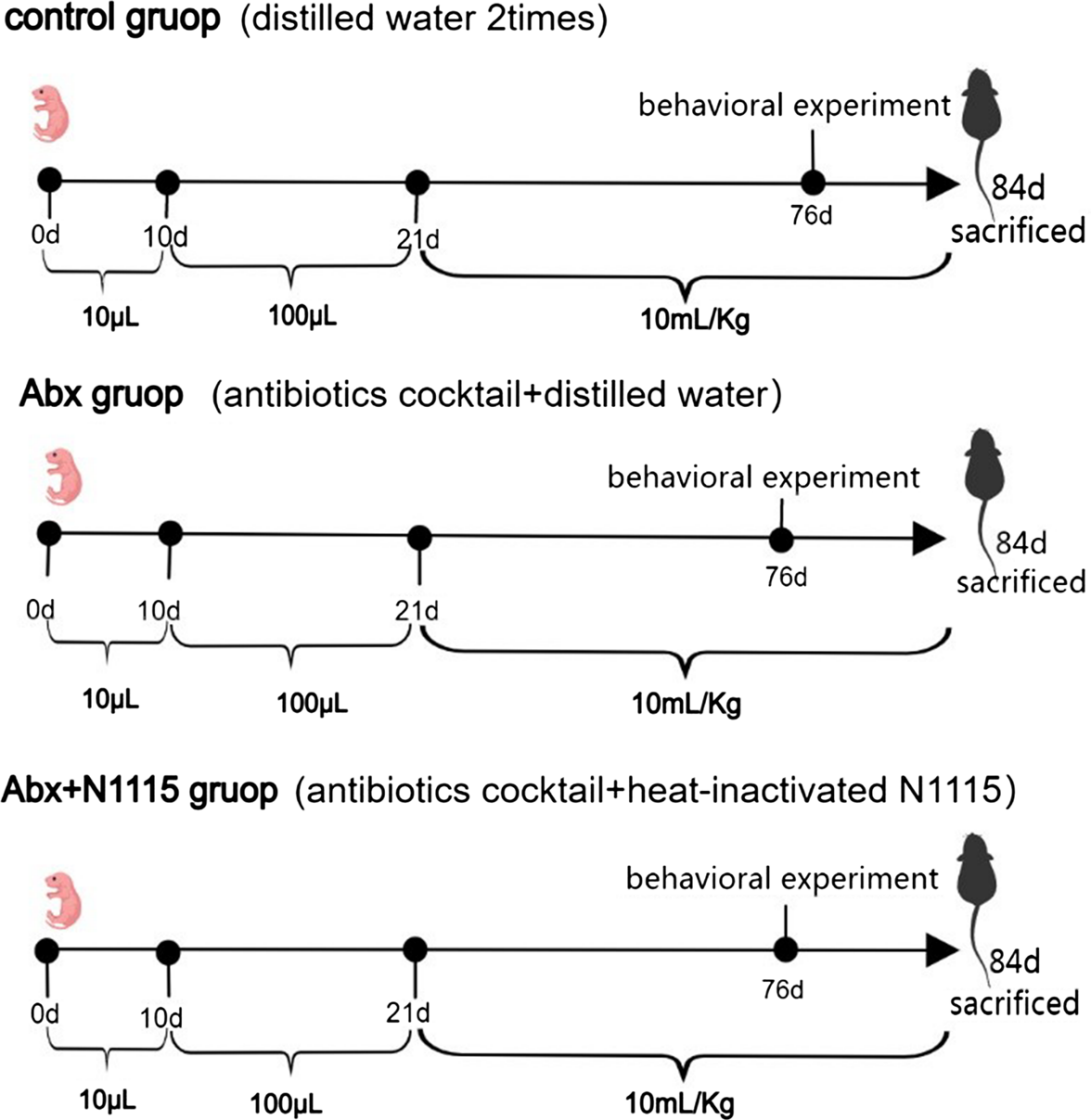
A protocol of experiment groups and treatment. The control group was gavaged with distilled water, and the Abx and Abx + N1115 groups were gavaged with the antibiotics cocktail. After two hours, the Abx + N1115 group was gavaged with 10^9^ CFU per mouse of heated-inactivated N1115, while other groups were gavaged with distilled water. All the mice were gavaged until PND21, and the behavioral experiments were carried out from PND76 to 84, and sacrificed at PND84

The antibiotics cocktail consisted of 100 mg/kg ampicillin, 50 mg/kg vancomycin, 100 mg/kg neomycin, 100 mg/kg bacitracin, 50 mg/kg imipenem and 1 mg/kg amphotericin B (Dalian Meilun Biotechnology, Dalian, China) [23]. *L. paracasei* N1115 was sponsored by Shijiazhuang Junlebao Dairy Co. Ltd. (Shijiazhuang, China). The bacterium preparation was dissolved in sterile saline and heated at 65 °C for 2 h. The heat-inactivated *L. paracasei* N1115 were resuspended in distilled water. The control group was gavaged with distilled water while the Abx and Abx + N1115 groups were gavaged with the antibiotics cocktail. After two hours, the Abx + N1115 group was gavaged with 10^9^ CFU of N1115 per mouse, and other groups were gavaged distilled water. The gavage volume for each mouse were 10 µL for postnatal days (PNDs) 0–10, 100 µL from PNDs 10**–**21, and 10 mL/kg from PNDs 21 to 84 [22]. Female mice were euthanized on PND21 and only male mice were maintained till the experiment ended (Fig. 1).

### Morris maze test

The Morris maze test was used to test the spatial positioning and memory of the mice. The water surface was divided into four equal quadrants (N, S, W, and E). A platform was placed in the center of the SW quadrant. During the test, the water temperature was maintained between 21 and 22 °C. During training time, the mice entered the water four times from different quadrants for 5 days at 1 min/time. The latency, which is the time for the mice to find the platform, was calculated as the average of the four times and limited to 60 s for this test. On the 6th day or test day, the mice went into the pool without a platform and the latency on this day was the first time point when the mice passed the original platform position [24].

### Tail suspension test

Tail suspension test was used to measure the depressive state of the mice. One-third of the mouse’s tail was fixed to a rod, which was hung over the center of the box turning the mice upside-down [25]. The mice were observed for 2 min before the recording. The time of activity and stillness within 4 min were recorded.

### Open field test

Open field test was used to measure exploratory behavior and anxiety of the mice. The mice were put into the middle compartment of the box and then recorded for 5 min. The total movement time and distance, horizontal and vertical score were recorded as described previously [26].

### Enzyme-linked immunosorbent assay (ELISA)

After euthanizing the mice, blood samples were collected and serum levels of interleukin-1β (IL-1β), interleukin-6 (IL-6), interleukin-10 (IL-10), tumor necrosis factor-α (TNF-α) and corticosterone were measured using ELISA kits (R&D Systems Inc., MN, USA) following the manufacturer’s instructions.

### Histopathology

The intestinal tissues were collected and fixed in formalin for 24 h and the ileum and colon tissues were stained using hematoxylin and eosin. The ileal villi lengths or the colonic crypts depths were measured in at last three field for each sample [22].

### Real time polymerase chain reaction (RT-PCR)

Tissues from the hippocampus and prefrontal cortex were collected and frozen at − 80 ℃.The mRNA expression levels of BDNF, γ-γ-aminobutyric acid type A receptor α1 (GABA_Aα1_), γ-aminobutyric acid type B receptor 1 (GABA_b1_), 5-hydroxytryptamine receptor 1 A (5-HT_1A_), mineralocorticoid receptor (MR), nerve growth factor (NGF), and glucocorticoid receptor (GR) were measured using reverse-transcription polymerase chain reaction (RT-PCR). Total RNA from the hippocampus and prefrontal cortex was extracted using TRIzol total RNA extraction kit (Chengdu Lanbo Biotechnology Co., Ltd. Chengdu, China). RT-PCRwas performed using the iScript™ gDNA Clear cDNA Synthesis Kit (Bio-Rad Laboratories, Berkeley, CA, USA).

The reaction mixture consisted of 5 µL of SsoFast EvaGreen supermix and 0.3 µL of forward and reverse primer. The PCR cycling condition was as follows: 95 °C for 30 s, 95 °C for 5 s, and Tm °C 5 s for 40 cycles. The dissolution curve was read from 65 to 95 °C.

β-actin (Sangon Biotech Co., Ltd., Shanghai, China, No. B661302) was used as the invariant control, and the mRNA levels were expressed as fold changes after normalization to β-actin. Primer sequences are listed in Table 1.

**Table 1:** Real-time PCR primers

Target	sequences	Amplicon(bp)	Tm(℃)
β-actin	Purchased from Sangon Biotech (Shanghai) Co., Ltd. Number: B661302	174	60
BDNF	F: 5′- TGG AAC TCG CAA TGC CGA ACT AC -3′ R: 5′- TCC TTA TGA ATC GCC AGC CAA TTC TC -3′	88	58
GABA_Aα1_	F: 5′- AAA AGT CGG GGT CTC TCT GAC - 3′ R: 5′- CAG TCG GTC CAA AAT TCT TGT GA -3′	138	58
GABA_b1_	F: 5′- ACG TCA CCT CGG AAG GTT G -3′ R: 5′- CAC AGG CAG GAA ATT GAT GGC - 3′	107	57
5-HT_1A_	F: 5′- TGG GCA ATC ACC GAC CCT AT -3′ R: 5′- TAC CCG TGG TCC TTG CTG AT -3′	179	55.5
MR	F: 5′- GAA AGG CGC TGG AGT CAA GT -3′ R: 5′- TGT TCG GAG TAG CAC CGG AA -3′	127	58
NGF	F: 5′- GCA GAA CCG TAC ACA GAT AGC AA -3′ R: 5′- GTC AAG GGA ATG CTG AAG TTT AGT - 3′	84	60
GR	F: 5′- AGC TCC CCC TGG TAG AGA C -3′ R: 5′- GGT GAA GAC GCA GAA ACC TTG - 3′	120	58

#### 16 S rRNA encoding gene sequencing and bioinformatics analysis

Fresh stool samples were collected and frozen at − 80 °C. Total DNA was extracted using TIANamo Stool DNA kits (Tiangen Biotech Co., Ltd., Beijing, China). The reaction of next-generation sequencing reaction was conducted as previously described [22].

The third and fourth hypervariable regions (V3-V4) of the DNA sequence were amplified and tested on 2% agarose gel and the screened PCR products were sequenced. The Agilent 2100 biochip analysis system was used to detect the size and to quantify the amplicon library. The original data was converted to FastQ sequences. The sliding window method was employed to filter bases and choose effective sequences for analysis. Sequences similarity of up to 97% was called operational taxonomic units (OTUs) and based on the OTUs and species, the α diversity index, principal coordinate analysis (PCoA), community structure of microbiota, and species abundance of different levels were analyzed.

### Statistical analysis

All statistical analyses were performed using SPSS 23.0 (SPSS, Inc., IL, USA) and the data was expressed as mean ± SD. One-way ANOVA and Kruskal–Wallis H-test were used for comparisons between groups, and Turkey test was used as the post hoc test. We used two-way ANOVA for analyzing Morris maze test. Statistical significance was considered at *P* < 0.05.

## Results

### Intestinal tissue damage

**Fig. 2:**
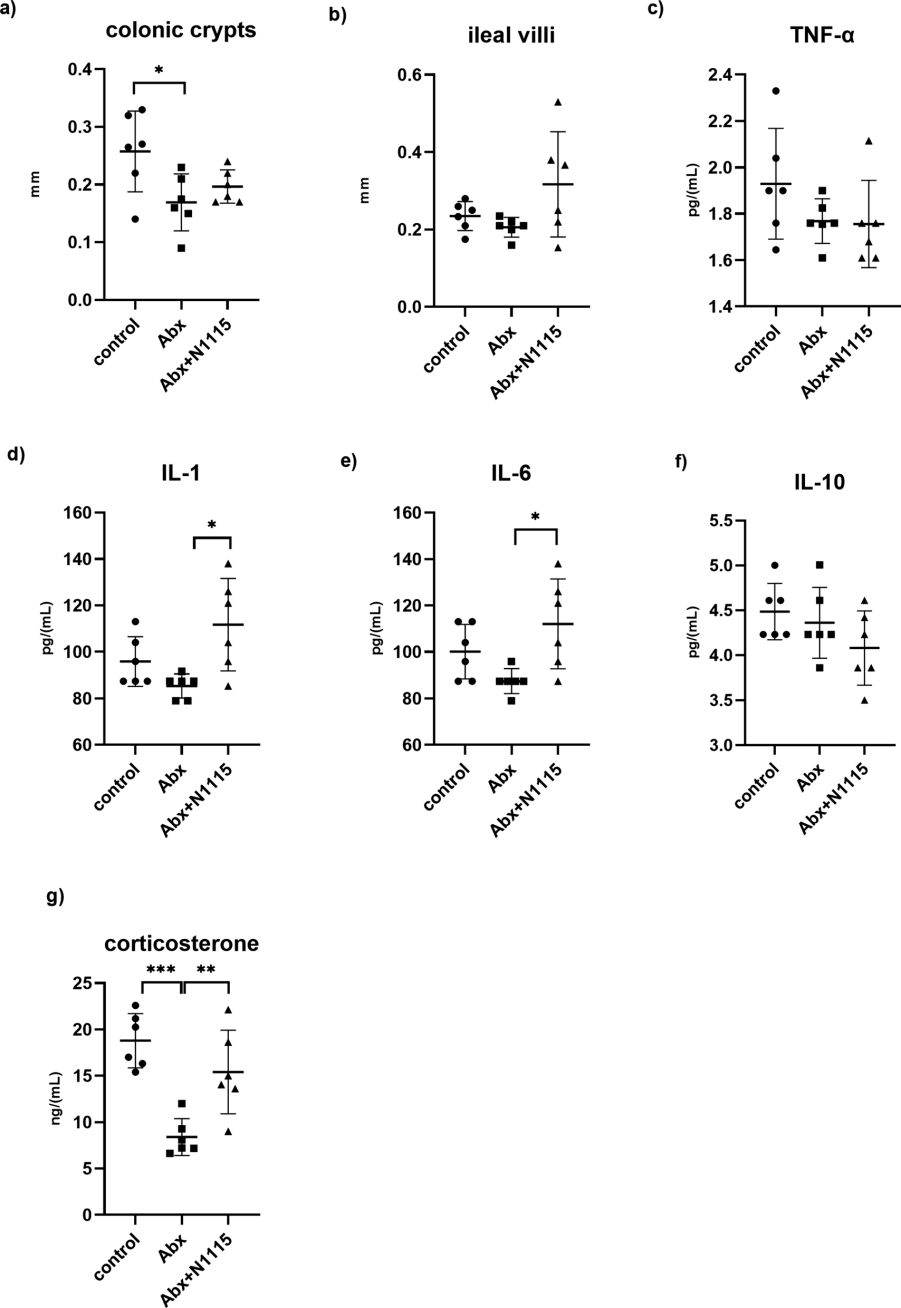
The effect of long-term intervention of antibiotics and heat-inactivated N1115 on intestinal tissue, serum cytokines and corticosterone. (n = 6). Pathological results of **a** colonic crypts and **b** ileal villi. level of **c** TNF-α, **d** IL-1, **e** IL-6, **f** IL-10, **g** corticosterone in serum. *P < 0.05, **P < 0.01, ***P < 0.001

One-way ANOVA found a significant effect of antibiotics treatment on depth of colonic crypts (F _2, 15_ = 4.503, *P =* 0.0294). Compared with the control group, the Abx group had shallower crypts (*P* = 0.0263) (Fig. 2a). There was no significant difference in length of ileal villi between the three groups (F _2, 15_ = 2.894, *P =* 0.0856) (Fig. 2b).

### Changes in serum cytokines and corticosterone

One-way ANOVA found significant effects of treatment on the level of IL-1 (F _2,15_ = 8.882, *P =* 0.0130), IL-6 (F _2,15_ = 5, *P* = 0.0213) and corticosterone (F _2, 15_ = 15.38, *P* = 0.0002). Compared with the Abx group, the Abx + N1115 group had a higher level of IL-1 (*P* = 0.0103) (Fig. 2d). Compared with the Abx groups, the Abx + N1115 group had a higher level of IL-6 (*P* = 0.0164) (Fig. 2e). Compared with the control and Abx + N1115 groups, the Abx group had a lower level of corticosterone (*P* = 0.0002, *P* = 0.0061) (Fig. 2g). There was no significant difference in the level of TNF-α (F _2, 15_ = 1.658, *P =* 0.2236) (Fig. 2c) and IL-10 (F _2, 15_ = 1.825, *P =* 0.1925) (Fig. 2f) between the three groups.

### Expression patterns of neural signaling-related molecules in the hippocampus and the prefrontal cortex

**Fig. 3:**
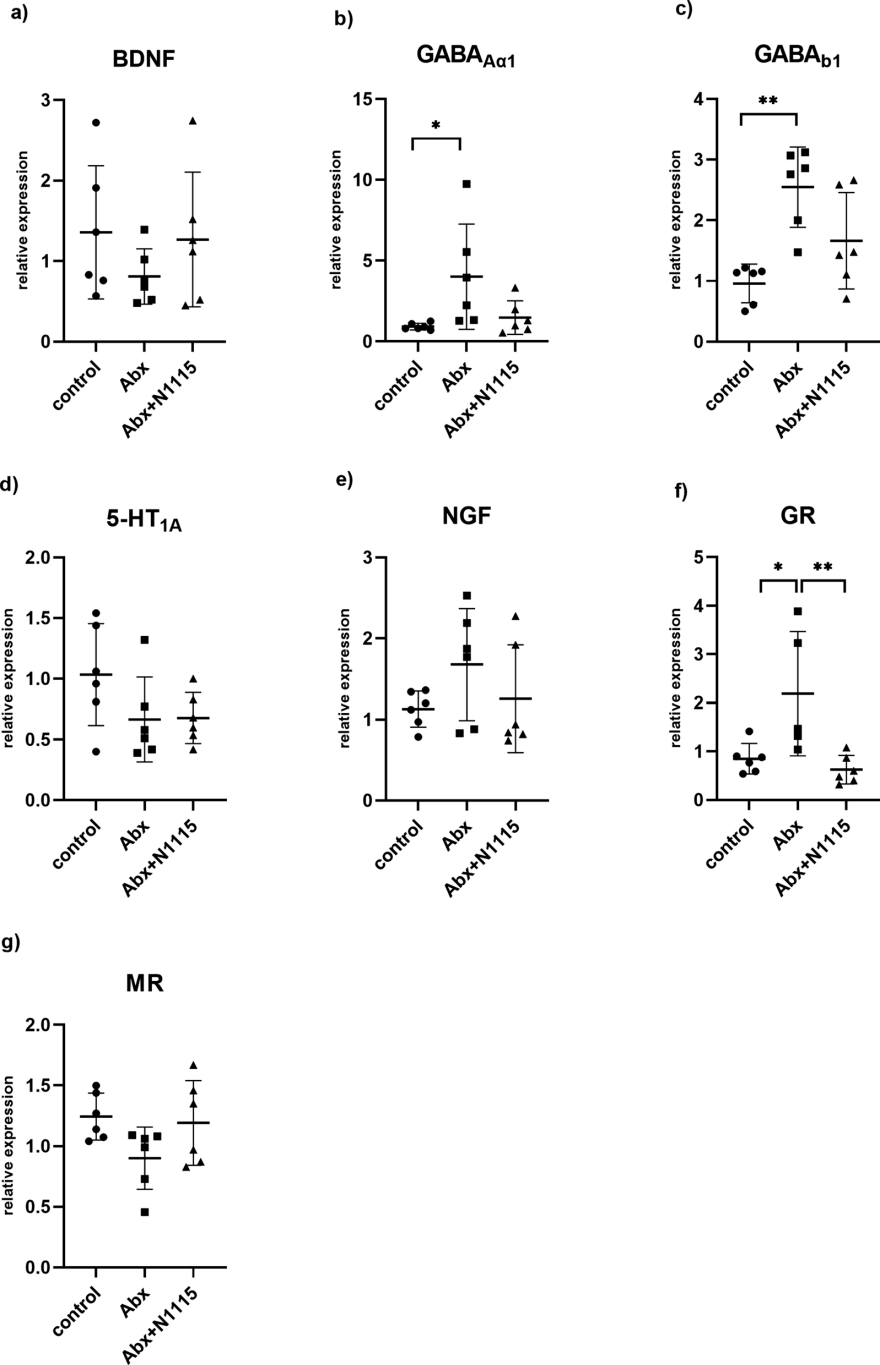
The effect of long-term intervention of antibiotics and heat-inactivated N1115 on brain function related molecular mRNA expressions of the hippocampus. (n = 6, GR of the control group n = 5). **a** BDNF, **b** GABA_Aα1_, **c** GABA_b1_, **d** 5-HT_1A_, **e** NGF, **f** GR, **g** MR. *P < 0.05, **P < 0.01, ***P < 0.001

As shown in Fig. 3, the mRNA expression levels of GABA_Aα1_, GABA_b1_, GR, BDNF, 5-HT_1A_, NGF, and MR in the hippocampus were measured. One-way ANOVA found significant effects of treatment on the mRNA expression levels of GABA_Aα1_(F _2, 15_ = 4.153 *P =* 0.0367), GABA_b1_ (F _2, 15_ = 9.724, *P =* 0.0020), and GR (F _2, 14_ =7.106, *P =* 0.0074).

Compared with the control group, the Abx group had higher expression levels of GABA_Aα1_ (*P* = 0.0409) (Fig. 3b) and GABA_b1_ (*P* = 0.0014) (Fig. 3c). Compared with the control and Abx + N1115 groups, the Abx group had a higher expression level of GR (*P* = 0.0231, *P* = 0.0087) (Fig. 3f). There was no significant difference in the expression levels of BDNF (F _2, 15_ = 1, *P =* 0.3782), 5-HT_1A_ (F _2, 15_ =2.329, *P =* 0.1316), NGF (F _2, 15_ = 1.536, *P =* 0.2472) and MR (F _2, 15_ = 2.739, *P =* 0.0969) between the three groups (Fig. 3a, d, e and g).

**Fig. 4:**
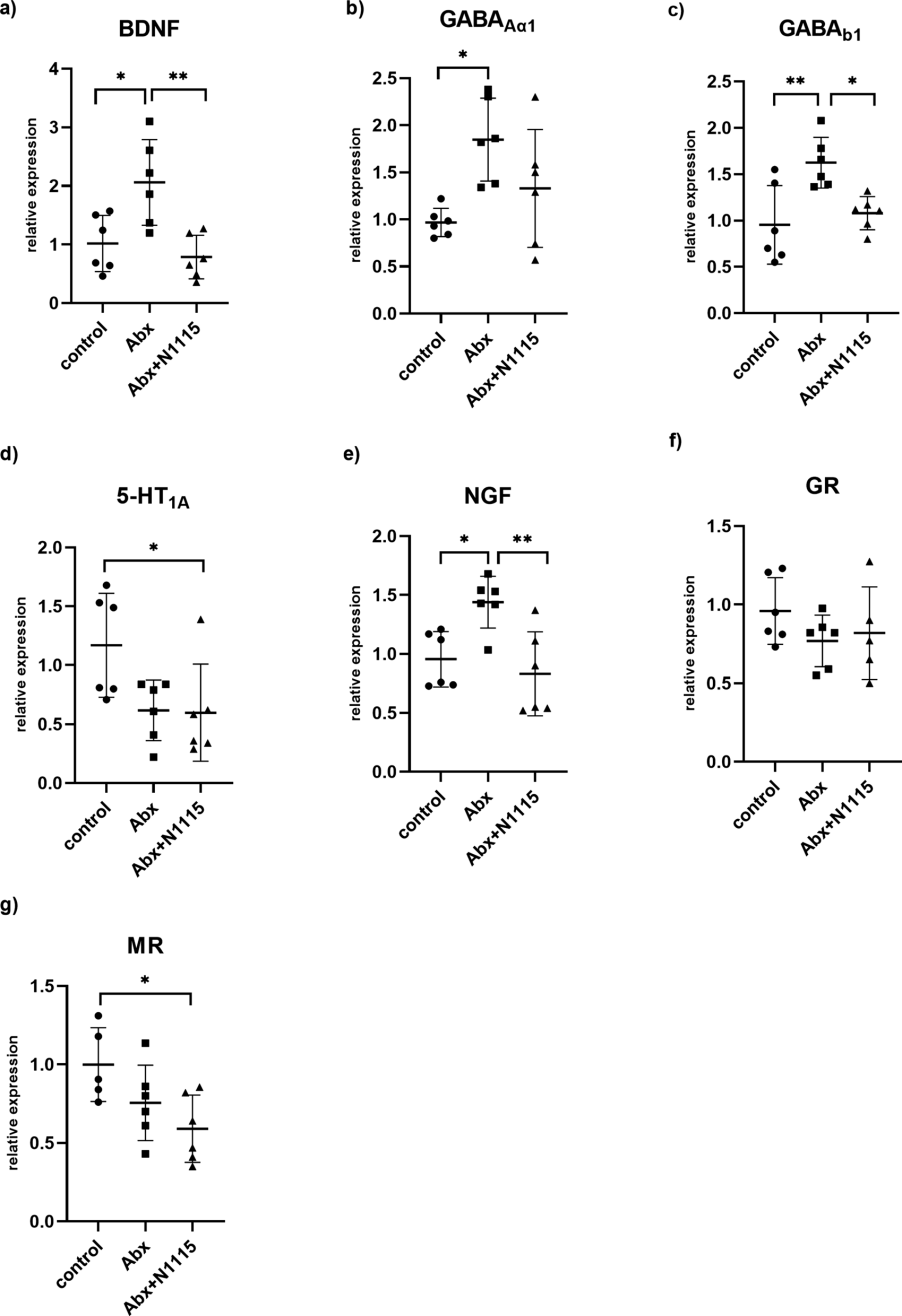
The effect of long-term intervention of antibiotics and heat-inactivated N1115 on brain function related molecular mRNA expressions of the prefrontal cortex. (n = 6, MR of the Abx + N1115 group and MR of the control group n = 5). **a** BDNF, **b** GABA_Aα1_, **c** GABA_b1_, **d** 5-HT_1A_, **e** NGF, **g** GR, **f** MR. *P < 0.05, **P < 0.01, ***P < 0.001

The mRNA expression levels of GABA_Aα1_, GABA_b1_, GR, BDNF, 5-HT_1A_, NGF, and MR in the prefrontal cortex were also measured and showed in Fig. 4. One-way ANOVA found significant effects of treatment on the mRNA expression levels of BDNF (F _2, 15_ = 9.171, *P =* 0.0025), GABA_Aα1_ (F _2, 15_ =5.785, *P =* 0.0137), GABA_b1_ (F _2, 15_ = 7.988, *P =* 0.0043), 5-HT_1A_ (F _2, 15_ = 4.421, *P =* 0.0309), NGF (F _2, 15_ =8.091, *P =* 0.0041) and MR (F _2, 14_ = 4.326, *P =* 0.0344).

Compared with the control and Abx + N1115 groups, the Abx group had higher mRNA expression levels of BDNF (*P* = 0.0129, *P* = 0.0030) (Fig. 4a) and GABA_b1_ (*P =* 0.0051, *P* = 0.0207) (Fig. 4c). Compared with the control group, the Abx group had a higher expression level of GABA_Aα1_ (*P =* 0.0107) (Fig. 4b). Compared with the control and Abx groups, the Abx + N1115 group had a higher expression level of NGF (*P* = 0.0216, *P* = 0.0046) (Fig. 4e). Compared with the control group, the Abx + N1115 group had lower expression levels of 5-HT_1A_ (*P* = 0.0478) (Fig. 4d) and MR (*P* = 0.0275) (Fig. 4 g). No significant difference in GR was found among the groups (F _2, 14_ = 1.151, *P =* 0.3446) (Fig. 4f).

### Morris water maze test

**Fig. 5:**
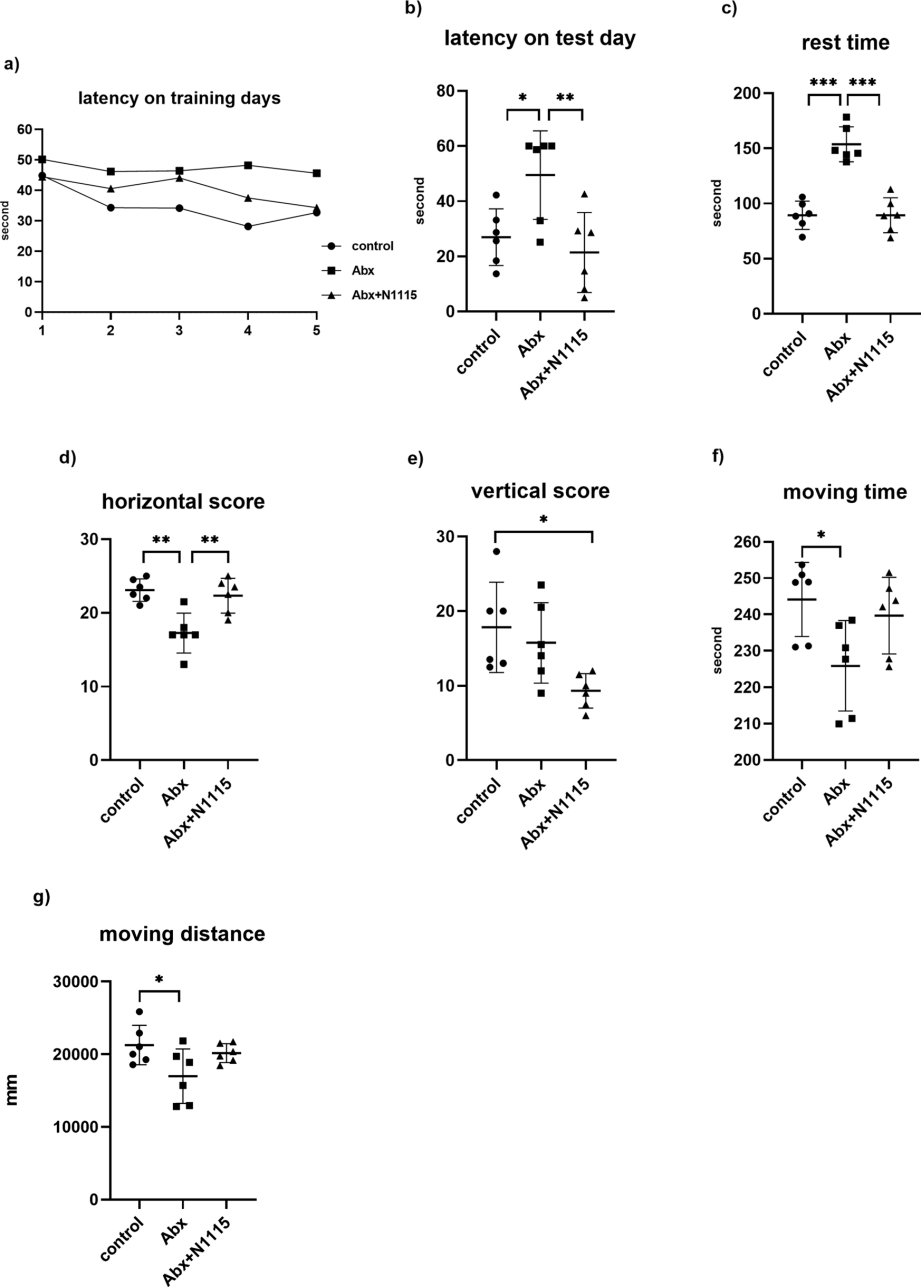
The effect of long-term intervention of antibiotics and heat-inactivated N1115 on behavioral test. (n = 6). Changes of **a** latency on training days and **b** latency on test day on Morris water maze test, and the maximum latency is 60s. Changes of **c** rest time on tail suspension test and **d** horizontal score, **e** vertical score, **f** moving time and **g** moving distance on open field test. *P < 0.05, **P < 0.01, ***P < 0.001

As shown in Fig. 5a, we found the latency was decreased with training days increased. Two-way ANOVA found the latency of mice was affected by treatments and training time. but there was no interaction effect between the two factors (F = 1.665, *P* = 0.089). After the same training period, the comparisons between groups only considered the effect of treatment. One-way ANOVA found a significant effect of treatment on the latency of the test day (F _2, 15_ = 6.959, *P =* 0.0073). Compared with the control and Abx + N1115 groups, the Abx group had longer latency (*P* = 0.0324, *P* = 0.0081) (Fig. 5b). No significant difference was found between the Abx + N1115 and control groups.

### Tail suspension test

One-way ANOVA found a significant effect of treatment on the rest time (F _2, 15_ = 37.26, *P* < 0.0001). Compared with the control and Abx + N1115 groups, the Abx group has a longer rest time (*P* < 0001, *P* **≤** 0001) (Fig. 5c). No significant difference in rest time was found between the Abx + N1115 and control groups.

### Open field test

One-way ANOVA found significant effects of treatment on the horizontal score (F _2, 15_ = 11.86, *P =* 0.0008), vertical score (F _2, 15_ =4.968, *P =* 0.0221), movement time (F _2, 15_ = 4.409, *P =* 0.0312) and movement distance (F _2, 15_ = 3.873, *P =* 0.0441). Compared with the Abx group, the control and Abx + N1115 groups had higher horizontal scores (*P =* 0.0012, *P* = 0.0038), and no significant difference was found between the Abx + N1115 and control groups (Fig. 5d). Compared with the control group, the Abx + N1115 had a lower vertical score (*P =* 0.0219) (Fig. 5e). Compared with the control group, the Abx group had less movement time (*P =* 0.0309), and no significant difference between the Abx + N1115 and control groups (Fig. 5f). Compared with the control group, the Abx group had less movement distance (*P =* 0.0472), and no significant difference between the Abx + N1115 and Abx groups (Fig. 5g).

### Alterations in gut microbiota diversity and composition

**Fig. 6:**
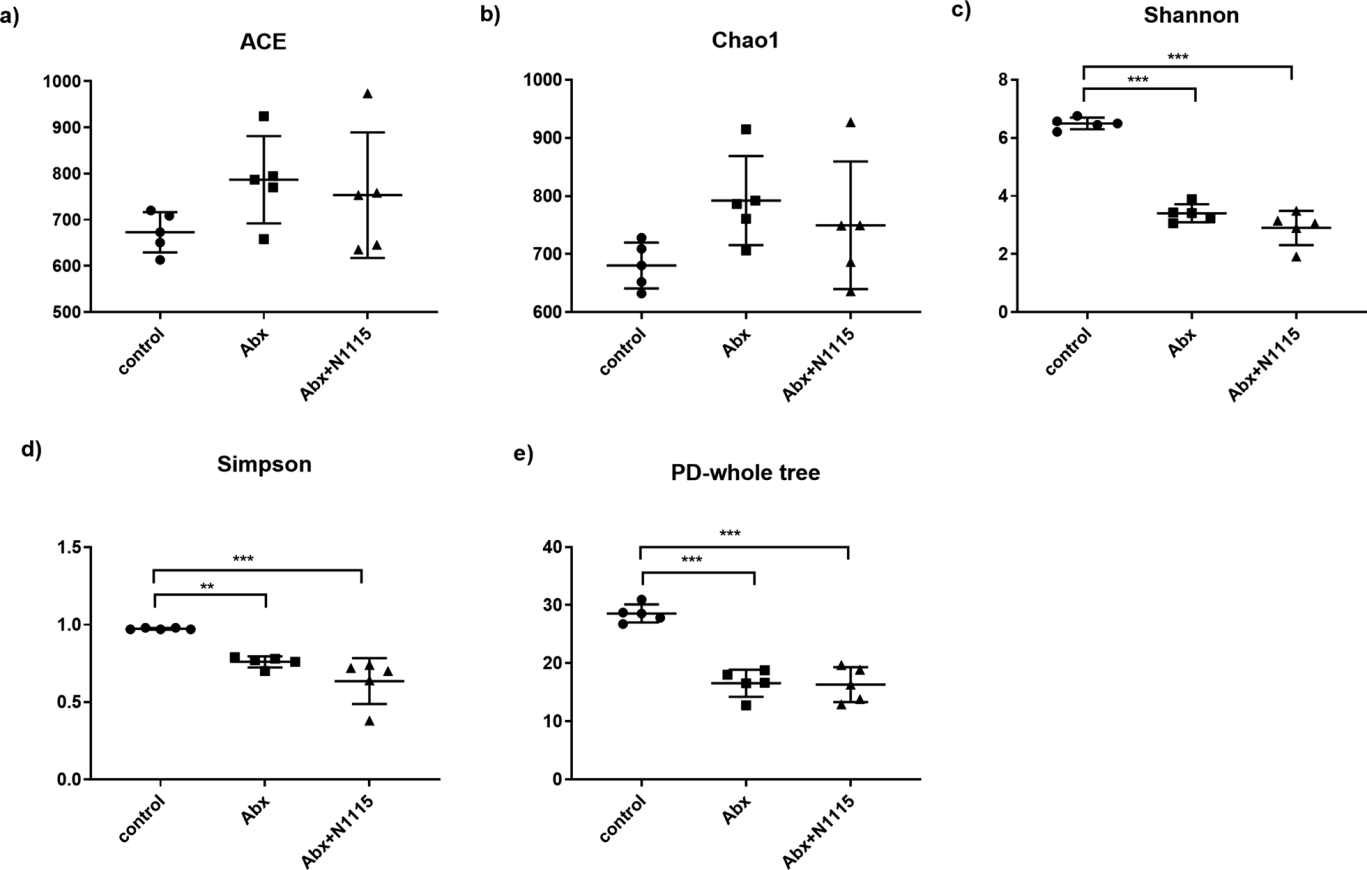
The effects of long-term intervention of antibiotics and heat-inactivated N1115 on α diversity of gut microbiota. (n = 5). **a** ACE index and **b** Chao1 index estimate the community richness of microbiota. **c** Shannon index and **d** Simpson index estimate the community diversity of microbiota. **e** PD-whole tree is a diversity index which is calculated based on the phylogenetic tree. The larger the value means the higher the community diversity. *P < 0.05, **P < 0.01, ***P < 0.001

**Fig. 7:**
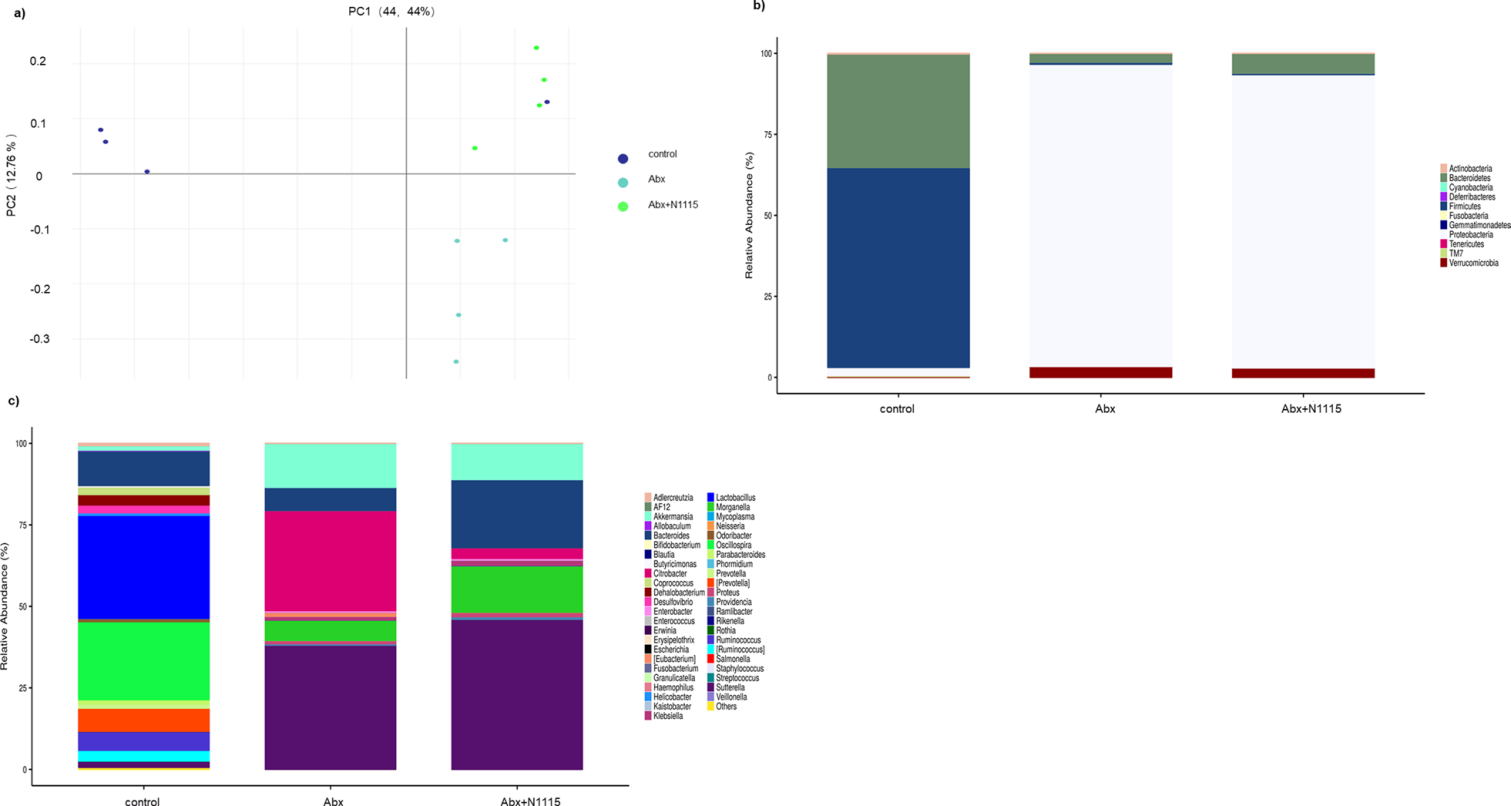
The effects of long-term intervention of antibiotics and heat-inactivated N1115 on structure of gut microbiota. **a **The PCoA analysis based on the weighted UniFrac distance. **b** Microbiota composition at the phylum and **c** genus levels. rare taxa (< 1%) are classifed into others

One-way ANOVA found significant effects of treatment on the Shannon (F_2, 12_ = 117.4, *P* < 0.0001), Simpson index (F_2, 12_ = 18.94, *P* < 0.0002) and the PD whole tree (F_2, 12_ = 43.85, *P* < 0.0001). Compared with the control group, the Abx and Abx + N1115 groups had lower Shannon index (*P* < 0.0001, *P* < 0.0001) (Fig. 6c), Simpson index (*P* < 0.0060, *P* < 0.0002) (Fig. 6d) and PD whole tree (*P* < 0.0001, *P* < 0.0001) (Fig. 6e). No significant difference was found between the ACE and Chao1 indexes (Fig. 6a and b). Treatment with antibiotics reduced the diversity of the microbiota. PCoA analysis based on the weighted UniFrac distance revealed significant differences in the gut microbial composition between all the groups. PC1 and PC2 contributed to 69.11% and 6.88% of these variabilities, respectively. PC1-based separation of the groups indicated that antibiotics majorly impacted the gut microbiota structure. Meanwhile, PC2 separated the Abx and Abx + N1115 groups, indicating that heat-inactivated N1115 could slightly alleviated antibiotic-induced damage (Fig. 7a).

The composition of the gut microbiota was also greatly altered. As shown in Table 2, the composition of gut microbiota differed at the phylum and genus levels in all groups. After long-term antibiotics intervention, the dominant phylum changed from *Bacteroides* to *Proteobacteria*. At the phylum level, *Proteobacteria* was more abundant in the Abx and Abx + N1115 groups while *Firmicutes* and *Bacteroidetes* were significantly less abundant than the control group (Fig. 7b) (Table 2).

**Table 2:** Mean relative abundance of different taxa at phylum and genus level

		Control (%)	Abx (%)	Abx + N1115 (%)
Phylum	*Proteobacteria*	2.55	93.27***	90.51***
	*Bacteroidetes*	61.69	0.63**	0.43**
	*Firmicutes*	34.98	2.71**	6.13*
	*Verrucomic*	0.26	3.37*	2.93
Genus	*Sutterella*	1.85	38.06***	46.04***^###^
	*Citrobacter*	< 0.01	30.7%***	3.29***^###^
	*Ruminococcus*	5.72	< 0.01**	0.01**
	*Coprococcus*	2.33	< 0.01**	< 0.01**
	*Oscillospira*	23.93	0.04**	0.01**
	*Lactobacillus*	31.65	0.04**	0.08**
	*Morganella*	0.04	6.2**	14.25***^#^
	*Klebsiella*	< 0.01	1.12**	1.55**
	*Prevotella*	1.35	< 0.01*	< 0.01*
	*Akkermansia*	1.39	13.49*	11.1
	*Proteus*	< 0.01	0.93	1.3*^#^

Compared with control group, * *P* < 0.05, **P < 0.01, ***P < 0.001

Compared with Abx group, ^#^ *P* < 0.05, ^##^ *P* < 0.01, ^###^ *P* < 0.001

At the genus level, *Sartreella*, *Citrobacter*, *Morganella*, and *Klebsiella* were significantly more abundant in the Abx and Abx + N1115 groups while *Rumenococcus*, *Oscillatoria*, *Lactobacillus* and *Prevotella* were less abundant in the Abx and Abx + N1115 groups than the control group. Further, the abundance of *Surtreella*, *Morganella*, and *Proteus* significantly increased but that of *Citrobacter* decreased in the Abx + N1115 group than the Abx group (Fig. 7c) (Table 2).

## Discussion

Paraprobiotics, which are the inactivated/dead/non-viable microbial cells of probiotics or crude cell extracts [27], have several health benefits. Our results showed that the negative effects of long-term exposure to antibiotics on the brain and the gut was substantially alleviated by heat-inactivated N1115 through the microbiota–gut–brain (MGB) axis.

We observed that N1115 significantly alleviated the long-term antibiotic cocktail-induced learning and memory dysfunction, anxiety and depression. Consistently, animal experiments and clinical studies have shown that while antibiotic exposure increases the risk of depression, and use of probiotics can improve cognitive and behavioral abnormalities and relieve depression [28–33]. Moreover, studies showed that probiotics reduced anxiety-like behavior in both rodents and patients [34–37]. In this study, we investigated the changes in the gut microbiota and the possible pathways involved in the MGB axis to elucidate how paraprobiotics alleviate cognitive functions and emotion.

Long-term antibiotic exposure significantly damages the intestinal tissues and gut microbiota structure. Studies have shown that the gut microbiota structure in patients with cognitive dysfunction or depression differs from that in healthy people. Changes in the gut microbiota are closely related to brain function. For example, *Sutterella* is more abundant in the intestines of children with autism [38–40]. *Citrobacter* is a pathogenic microorganism that can cause intestinal epithelial hyperplasia and colitis in mice and studies confirmed that this infection can be alleviated by probiotics [41–43]. The relative abundance of *Ruminococcus*, which is a genus of anaerobic, Gram-positive bacteria in the class of Clostridia and is closely associated with Crohn’s disease [44], is significantly reduced in patients with PD [45, 46]. We have shown that heat-inactivated N1115 significantly reduced the relative abundance of *Citrobacter* while increased that of *Ruminococcus*. These results indicated that the paraprobiotics, such as heat-inactivated N1115, could alleviate the gut microbiotal disorder caused by long-term exposure to antibiotics but cannot enhance colonization in the intestinal tract.

Studies have proposed that the common pathways of the MBG axis are the immune system, endocrine system, neurotransmitter, and HPA axis. The immune system is one of most common ways in the MGB axis [47, 48]. IL-6 and IL-1β are pro-inflammatory factors while IL-10 reduces inflammatory response by downregulating IL-1 and TNF-α levels [49]. In this study, heat-inactivated N1115 significantly upregulated IL-1β and IL-6 levels in the Abx + N1115 group. There was a systematic review study found that some probiotics as exogenous substances to cause infections in children [50]. In our study, the long-term use of antibiotics suppressed the immune system, and we suspect that heat-inactivated N1115 as an exogenous antigen that stimulated the immune system and enhanced the immune response. Compared with probiotics, heat-inactivated N1115 could not colonize and migrate so that did not cause invasive infections.

The composition of the HPA axis, corticosterone, and its receptors, GR and MR, were also significantly altered by long-term antibiotic exposure that affects different brain regions. However, heat-inactivated N1115 effectively regulated the antibiotic-induced abnormalities via the HPA axis. We observed differential expressions of functional genes in different brain regions. N1115 also restored abnormally higher mRNA levels of GABA_Aα1_ and GABA_b1_ in the hippocampus and prefrontal cortex after using antibiotics back to normal levels. While the mRNA expression levels of BDNF, NGF, and 5-HT_1A_ were upregulated in the prefrontal cortex after using antibiotics, this was not observed in the hippocampus. These results showed that the effect of antibiotics and inactivated N1115 on the cognitive function through the MGB axis is more significant in the prefrontal cortex than in the hippocampus. The hippocampus is related to memory, while the prefrontal cortex is associated with emotions [51, 52]. 5-HT and GABA receptors can be used as targets for anti-anxiety drugs [53]. This finding also suggests that inactivated N1115 improves behavior and cognitive function by regulating these neurotransmitter receptors, acting as a mood drug.

Several studies confirmed the benefits of probiotics on brain function, but only few studies focused on paraprobiotics. Studies have found that several bioactive compounds from inactivated probiotic cells, such as peptides, proteins, and peptidoglycans, can regulate gut microbiota, immune function and HPA axis [54–57]. The immune and HPA axis are important pathways linking gut microbiota and brain function. Here we also found that the gut microbiota composition and certain biomolecules related to immunity and HPA axis, such as IL-1, IL-6 and corticosterone were significantly altered after using heat-inactivated N1115. Therefore, we suggest that heat-inactivated N1115 might alleviate the side effects of antibiotics on the brain by regulating the gut microbiota, immune system, HPA axis, and brain function-related gene expression through its bioactive compounds.

## Data Availability

The data from the next-generation sequencing in this study can be found in NCBI BioProject database under accession number PRJNA846941. The SRA records will be accessible with the following link: https://www.ncbi.nlm.nih.gov/sra/PRJNA846941. The other datasets generated and analysed during the current study are not publicly available du the study has not been fully disclosed as a postgraduate graduation project, but are available from the corresponding author on reasonable request.
